# Studies on alterations of clinical and hemato-biochemical parameters before and after treatment in calves naturally infected with theileriosis

**DOI:** 10.14202/vetworld.2016.1381-1385

**Published:** 2016-12-07

**Authors:** J. P. Kachhawa, Surender Kumar, Ankita Sharma, A. P. Singh, Anil Ahuja

**Affiliations:** 1 Department of Clinical Veterinary Medicine, Ethics and Jurisprudence, College of Veterinary & Animal Science, Rajasthan University of Veterinary and Animal Sciences, Bikaner - 334 001, Rajasthan, India; 2 Veterinary Hospital, Haripura, Hanumangarh, Rajasthan, India

**Keywords:** blood transfusion, buparvaquone, calves, hemato-biochemical, theileriosis

## Abstract

**Aim:**

The aim was to determine hemato-biochemical alterations and to determine the better treatment of theileriosis in naturally infected calves.

**Materials and Methods:**

A total of 74 Holstein crossbred calves below 6 months of age, of either sex were included for present investigation in Bikaner. Based on the clinical examinations and laboratory results, 20 calves included for hemato-biochemical studies (before and after treatment) and divided into two groups (having 10 calves each).

**Results:**

The clinical examination of these calves revealed weakness, ticks infestations, high fever above 104°F, pronounced swelling of prescapular, prefemoral, parotid lymph nodes, loss of elasticity of skin, anemia, lacrimal discharges, pulpy cornea, tachycardia, and dyspnea. There was highly significant decrease (p<0.01) in hemoglobin, total erythrocyte count, packed cell volume and total leukocyte count, serum glucose, total protein, globulin and albumin level and highly significant increase in aspartate aminotransferase and alanine aminotransferase level as compared to healthy control animals in Group I and II. The animals of Group II treated with buparvaquone along with single blood transfusion shows better recovery then animals of Group I treated with bupavaqone and hematinic.

**Conclusions:**

Significant changes were found in hemato-biochemical parameters in theileria affected calves before treatment as compare to healthy control calves. Significant improvement was observed in hemato-biochemical parameters in buparvaquone and single blood transfusion treated calves as compare to another group, so it is concluded that buparvaquone and single blood transfusion is better combination for treatment of theileriosis.

## Introduction

Tropical theileriosis is a major tick-borne protozoan disease of cattle and is associated with high morbidity and mortality. However, indigenous cattle (*Boss indicus*) are less affected by this disease than crossbred cattle. It is mainly seen in cattle, sheep, and goats as well as in wild and captive ungulates [1-3]. Bovine tropical theileriosis is caused by *Theileria annulata* is an important disease of cattle in India and is mainly transmitted by tick *Hyalomma anatolicum anatolicum*.

*Theileriosis* causes heavy economic losses in terms of high morbidity, mortality and reduced production in recovered animals [4]. Around 10 million cattle are at risk for tropical theileriosis with an annual economic loss of around US $800 million in India [5]. The mortality rate with *T. annulata* may reach 70% with case fatality rate of 10-20% in calves while due to *Theileria parva* the mortality rate of 100% has been recorded in exotic cattle [6]. In many epidemiological situations, high mortality occurs only in calves; the adult cattle represent immune survivors [7]. Tentative diagnosis of theileriosis in the field is mainly based on clinical signs and tick infestation on the infected animals. However, confirmation of the diagnosis depends on microscopic examination of Giemsa-stained thin blood smears [8,9]. The demonstration of Koch’s blue bodies in the lymphocytes and monocytes of the lymph node smear or peripheral blood film is pathognomonic of the disease [10].

The aim of the present study was to determine hemato-biochemical alterations and to determine the better treatment of theileriosis in naturally infected calves.

## Materials and Methods

### Ethical approval

The study was not conducted on experimental animals. We have taken cases from routine cases reported in clinic, so no need to take ethical approval. However, animals were examined and samples were collected as per standard examination and sample collection procedure.

### Selection of animals

For the present investigation, a total of 74 Holstein crossbred calves below 6 months of age, of either sex were included during June to October-2015 belonging to outdoor patients of Teaching Veterinary Clinical Complex, College of Veterinary and Animal Science, Bikaner.

Based on the clinical examinations and laboratory results, 22 calves were diagnosed as positive for theileriosis and divided into two groups, i.e., Groups I and II. Two calves were died from Group I during treatment so excluded from study, rest of 20 calves included for hemato-biochemical studies (before and after treatment) and divided into two groups (having 10 calves each). For comparison of hemato-biochemical status of clinically positive theileriosis infected calves, a total of 10 apparently healthy calves from organized privet dairy farm of Bikaner, were included in this study.

### Clinical observations

Clinical examination included history of ticks infestation, duration of illness, changes in managemental and feeding practice, appetite of the animal, abnormalities in the behavior, gait, posture, rumination, defecation (quantity, consistency and frequency), urination, examination of visible mucous membranes, eyes, skin and anus and general clinical examinations including rectal temperature, pulse, respiration and auscultation of heart and lungs [1].

### Hematological studies

Blood was collected in sterile tubes coated with disodium salt of ethylenediaminetetraacetic acid as an anticoagulant. The blood samples were subjected for determination of hemoglobin (Hb), packed cell volume (PCV), total erythrocyte count (TEC), and total leukocyte count (TLC) of the calves suffering from theileriosis and apparently healthy calves. These parameters were analyzed as per standard methods [11].

### Biochemical studies

For biochemical studies, blood was collected in sterile tubes without anticoagulant on day 0 as pre-treatment and on the 10^th^ day as post-treatment. The blood slants were made and incubated for 1 h at 37°C. Blood clots were broken, and tubes were centrifuged at 2500 rpm for 30 min. The serum was pipetted out in small Pyrex tubes and was kept immediately in the deep freeze at −20°C till analysis and was used for estimation of various biochemical parameters like total protein, albumin (Alb), globulin (Glb), glucose, aspartate aminotransferase (AST), and alanine aminotransferase (ALT), using commercially available kits of SPINREACT, S.A. - Ctra. Santa Coloma, 7-E-17176 SANT ESTEVE-(Girona), Spain.

### Treatment trials

A total of 20 positive cases of bovine tropical theileriosis infected Holstein crossbred calves were selected for treatment and classified into two groups (Group I and Group II having 10 calves each) irrespective of sex and included for pre- and post-treatment hemato-biochemical studies. Group I was given buparvaquone at the dose rate of 2.5 mg/kg b.wt. intramuscular. All the cases were given injection oxytetracycline at the dose rate of 10 mg/kg body weight intramuscular for 5 days and injection analgin at the dose rate of 1.5 g per calf and supportive treatment with injection B-complex, each ml contains methylcobalamin 500 mcg, pyridoxin 50 mg, nicotinamide 50 mg were given to each calf intramuscularly as per requirement and hematinic, contains each 21 g contains: Malt extract - I.P. 4.52, calcium guconate - I.P. 360 mg, ferric ammonium citrate - 30 mg (Eq.to 64.5 mg of Fe), copper sulfate - 100 mg, cobalt chloride - 1.5 mg, cholecalciferol - I.P. 3600 IU, nicotinamide - I.P. 45 mg, biotin - B.P. 75 mcg, folic acid - I.P 1.5 mg, cynocobalamin - I.P 15 mcg, flaored base q.s., color Caramel I.P. was given at the dose rate of 2 tsf as electuary to all calves.

Calves of Group II were given buparvaquone at the dose rate of 2.5 mg/kg b.wt. intramuscular. Single blood transfusion in required dose rate was performed in every infected calf. Whole blood was transfused in anemic calves was carried out [1,12,13]. While injection analgin at the dose rate of 1.5 g per calf and supportive treatment with injection B-complex were given to each calf intramuscularly as per requirement.

### Statistical analysis

All statistical data were analyzed using the basic statistics paired Student’s t-test by WASP [14], a web-based agricultural statistics software package.

## Results and Discussion

Based on laboratory test results 20 calves were diagnosed as positive for bovine tropical theileriosis from 74 calves during June to October-2015 was studied. The incidence can vary depending on the age of animal, breed, climatic conditions of the region, tick infestations, and management practices.

### Clinical manifestations

All the 22 calves infected with bovine tropical theileriosis were reported to be sick from last few days with additional history of inappetance or total refusal to suckle or feed. The clinical examination of these calves revealed weakness, ticks infestations, high fever above 104°F, unilateral or bilateral pronounced swelling of prescapular, prefemoral, parotid lymph nodes [1], loss of elasticity of skin, pale or anemic visible mucous membrane [15,16], lacrimal discharges, bilateral exophthalmia with dry and pulpy cornea [17], swelling of knee joints, tachycardia, dyspnea [18], pasty or bloody diarrhea, tachypnea, and dyspnea in few calves [19,20].

There was highly significant increase (p<0.01) in the mean±standard error (SE) value (Table-1) of body temperature, heart rate and respiration rate in bovine tropical theileriosis infected calves in Groups I and II as compared to healthy calves. The suggestive cause is that the cytokines like, tumor necrosis factor-α and interleukins (TNF-α, IL-1, and IL-6) produced by infected mononuclear cells are responsible for the diverse clinical symptoms of tropical theileriosis [21,22].

**Table-1:** Mean±SE value of clinical parameters in apparently healthy and bovine tropical theileriosis infected calves of Groups I and II (before and after treatment).

Parameters	Healthy calves (n=10)	Group I (n=10)		Group II (n=10)	
	
		Before treatment	After treatment	Before treatment	After treatment
Body temperature** (°F)	101.56±0.19^a^	105.67±0.16^b^	101.54±0.17^a^	105.84±0.24^b^	101.47±0.16^a^
Heart rate/min**	107.1±1.58^a^	140±2.91^b^	112.20±2.41^a^	146.7±3.08^b^	111.4±1.88^a^
Respiration rate/min**	23.6±0.93^a^	40.7±1.66^b^	26.4±1.30^a^	47.4±1.63^b^	24.9±1.00^a^

** p<0.01. Means with different superscripted letters in the same row differ significantly. SE=Standard error

### Hematological parameters

In this study, highly significant (p<0.01) decrease in mean±SE values of pre-treatment value of Hb, TEC, PCV and TLC level in bovine tropical theileriosis infected calves in Groups I and II as compared to healthy calves (Table-2). Earlier, similar findings were also recorded by several workers [23-25]. Although, some workers also observed significant negative correlations between parasitemia and both red blood cell count and PCV in theileriosis infected cattle [26].

**Table-2:** Mean±SE value of hematological parameters in apparently healthy and bovine tropical theileriosis infected calves of Groups I and II (before and after treatment).

Parameters	Healthy calves (n=10)	Group I (n=10)		Group II (n=10)	
	
		Before treatment	After treatment	Before treatment	After treatment
Hb (g/dl)**	11.67±0.3^a^	5.02±0.27^b^	5.86±0.17^c^	4.39±0.18^b^	7.39±0.20^c^
TEC (10^12^/L)**	6.57±0.30^a^	3.32±0.22^b^	4.41±0.27^c^	3.49±0.23^b^	5.16±0.22^c^
PCV (%)**	32.98±0.80^a^	17.13±0.74^b^	22.43±0.63^c^	16.80±0.46^b^	25.10±0.53^c^
TLC (10^9^/L)**	6.57±0.24^a^	4.07±0.12^b^	4.67±0.15^c^	4.51±0.16^b^	5.74±0.23^c^

** p<0.01. Means with different superscripted letters in the same row differ significantly. Hb=Hemoglobin, PCV=Packed cell volume, TEC=Total erythrocyte count, TLC=Total leukocyte count, SE=Standard error

There was highly significant increase (p<0.01) in Hb, TEC, PCV and TLC level as compared to its pre-treatment level in Group I and II. The decline in the values of Hb, TEC, and PCV may be attributed to the destruction of piroplasm infected erythrocytes by macrophages in the lymph nodes, spleen and other organs of the monocyte macrophage system coupled with reduced erythropoietic activity [23,27-29], the toxic effects of metabolites of *Theileria* species, persistent loss of blood caused by permanent bloodsucking ticks [30] and TNF-α on erythrogenesis [31].

In this study, leukopenia was seen in infected animals could have resulted from large scale destruction of lymphocytes by schizogony in lymphoid organs and infiltration of these cells into various organs, resulting in a decrease count in the peripheral circulation [32-34].

### Biochemical parameters

There was highly significant decrease (p<0.01) in mean±SE pre-treatment values of serum total protein, Glb, Alb, glucose and highly significant increase in AST and ALT level in bovine tropical theileriosis infected calves in Groups I and II as compared to healthy calves (Table-3). These findings are in accordance with several workers [23,26,29,35,36]. Although, the mean±SE values of serum total protein, Glb, Alb and glucose after treatment in Groups I and II were significantly lower as compared to healthy control. The decreased glucose serum concentration may be due to the abnormalities in liver functions and/or abnormalities in metabolism and anorexic state of affected animals [34,37,38]. The decreased serum total protein concentration, hypoalbuminemia and hyperglobulinemia could be explained that in naturally infected cattle with *T*. *annulata* causes liver failure [35,39]. Moreover, extra-vascular proteinaceous fluid in body cavities due to diseased lymph nodes [36,40], anorexia and diarrhea also cause hypoproteinemia [23,41]. There was highly significant decrease after treatment were observed in serum ALT and AST level in Groups I and II as compared to its pre-treatment level in all diseased groups.

**Table-3:** Mean±SE values of biochemical parameters in apparently healthy and bovine tropical theileriosis infected calves (before and after treatment).

Parameters	Healthy calves (n=10)	Group I (n=10)		Group II (n=10)	
	
		Before treatment	After treatment	Before treatment	After treatment
TP (g/dl)**	6.54±0.32^a^	4.66±0.17^b^	5.84±0.14^c^	5.04±0.17^b^	6.36±0.22^c^
Glb (g/dl)**	3.64±0.12^a^	2.77±0.14^b^	3.01±0.11^b^	2.97±0.16^b^	3.49±0.49^c^
Alb (g/dl)**	2.90±0.22^a^	1.88±0.17^b^	2.73±0.11^c^	2.49±0.08^a^	2.92±0.11^c^
Glucose (mg/dl)**	50.51±0.92^a^	33.24±0.82^b^	47.14±1.52^a^	33.04±1.49^b^	47.58±1.72^a^
ALT (IU/L)**	27.60±2.40^a^	70.37±6.41^b^	53.37±6.54^c^	81.70±6.59^b^	44.33±4.96^c^
AST (IU/L)**	44.30±2.64^a^	106.44±6.17^b^	72.04±6.69^c^	121.76±6.58^b^	84.65±6.39^c^

** p<0.01. Means with different superscripted letters in the same row differ significantly. Alb=Albumin, Glb=Globulin, AST=Aspartate aminotransferase, ALT=Alanine aminotransferase, TP=Total protein

Serum AST and ALT concentrations are the indicators of hepatic function [21] and are involved in amino acid and carbohydrade metabolism. *T. annulata* infection cause hepatic tissue damage that includes coagulative necrosis, distortion of hepatic cords and heavy infiltration of lymphocytes in the periportal areas [34].

### Group-wise comparison of treatment trial

Comparative efficacy of treatment in Groups I and II was seen with regard to clinical, hematological and biochemical parameters before and after treatment.

Out of 12 cases in Group I, two calves died during treatment. Seven cases received only single dose of injection buparvaquone. However, buparvaquone was repeated 48 h after first dosing in 3 calves. While no mortality observed in Group II. In the present investigation, the cure rates of Group I (buparvaquone and hematinics) and Group II (buparvaquone and single blood transfusion) were 83.33% and 100%, respectively.

Buparvaquone is a second generation hydro- xynaphthoquinone which inhibits the electron transport chain system of protozoa and not that of the host [42,43]. Several clinician successfully used buparvaquone as a drug of choice for the treatment of theileriosis in cattle alone [44,45] or in combination of oxytetracycline [16,46].

## Conclusion

The altered mean±SE values of hemato-biochemical parameters after treatment in Group I and II were near to the healthy control. However, the value attained in Group II was nearer to the healthy control with respect to other groups, indicated better recovery so buparvaquone and single blood transfusion is better combination for treatment of theileriosis.

## Authors’ Contributions

JPK and SK designed and planned the study. JPK and AS collected the samples and prepared the onsite smears slides. JPK, APS, and AS performed clinical, hemato-biochemical and treatment part of the study. APS and AA drafted the manuscript. All authors read and approved the final manuscript. JPK finalized the manuscript and sent for publication.
